# Supplementary material for: Ambient-Pressured Acid-Catalysed Ethylene Glycol Organosolv Process: Liquefaction Structure–Activity Relationships from Model Cellulose–Lignin Mixtures to Lignocellulosic Wood Biomass
Source: Polymers (Basel). 2021 Jun 17;13(12):1988. doi: 10.3390/polym13121988 (PMC8234995; doi:10.3390/polym13121988)
Supplement: Supplementary file 1 [file polymers-13-01988-s001.zip › polymers-1256978-supplementary.pdf]

Supporting Information

Ambient-pressured Acid-catalysed Ethylene Glycol Organosolv Process: Liquefaction Structure–Activity Relationships from Model Cellulose–Lignin Mixtures to Lignocellulosic Wood Biomass

Edita Jasiukaitytė-Grojzdek,* Filipa A. Vicente,* Miha Grilc and Blaž Likozar

Department of Catalysis and Chemical Reaction Engineering, National Institute of Chemistry, Hajdrihova 19, 1000 Ljubljana, Slovenia

***Co-corresponding authors**

E-mail addresses: edita.Jasiukaityte@ki.si and filipa.andre.vicente@ki.si

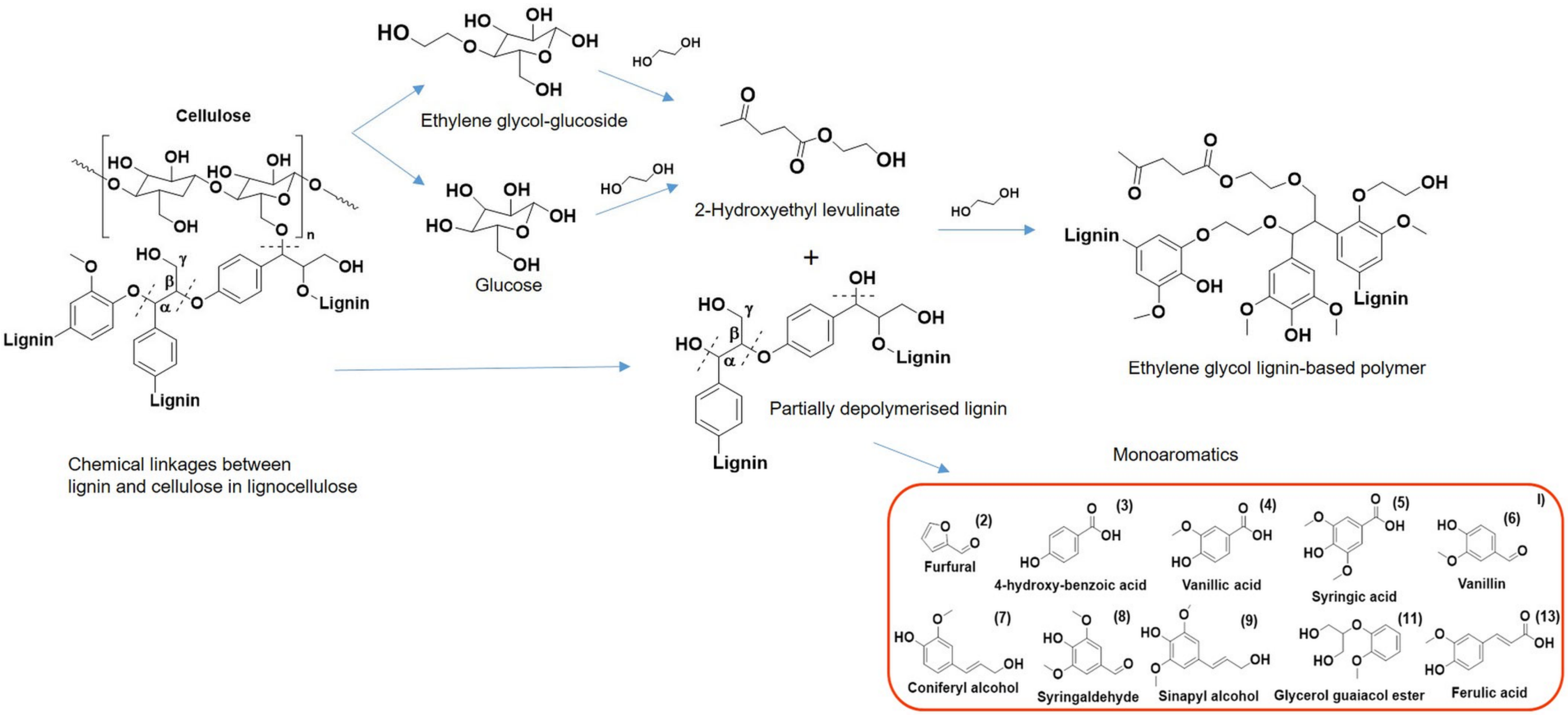

Figure S1. A tentative mechanism of the ethylene glycol lignin-based polymer and lignocellulose derived monomer formation.

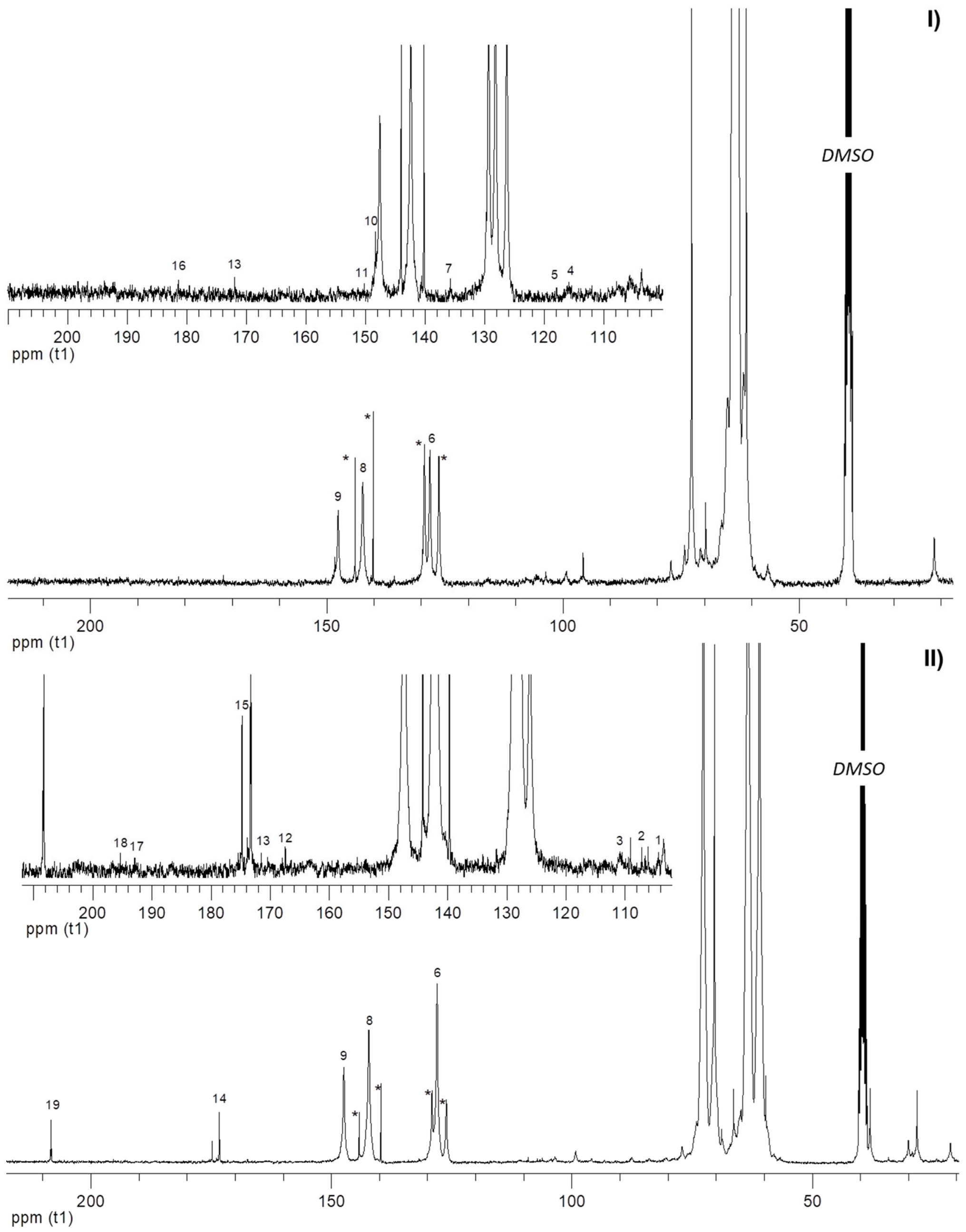

Figure S2. Qualitative ^{13}C NMR spectrum of **I)** CL$_{LGS}$-5 and **II)** CL$_{LGS}$-240; signals indicated with an asterisk (*) correspond to PTSA.

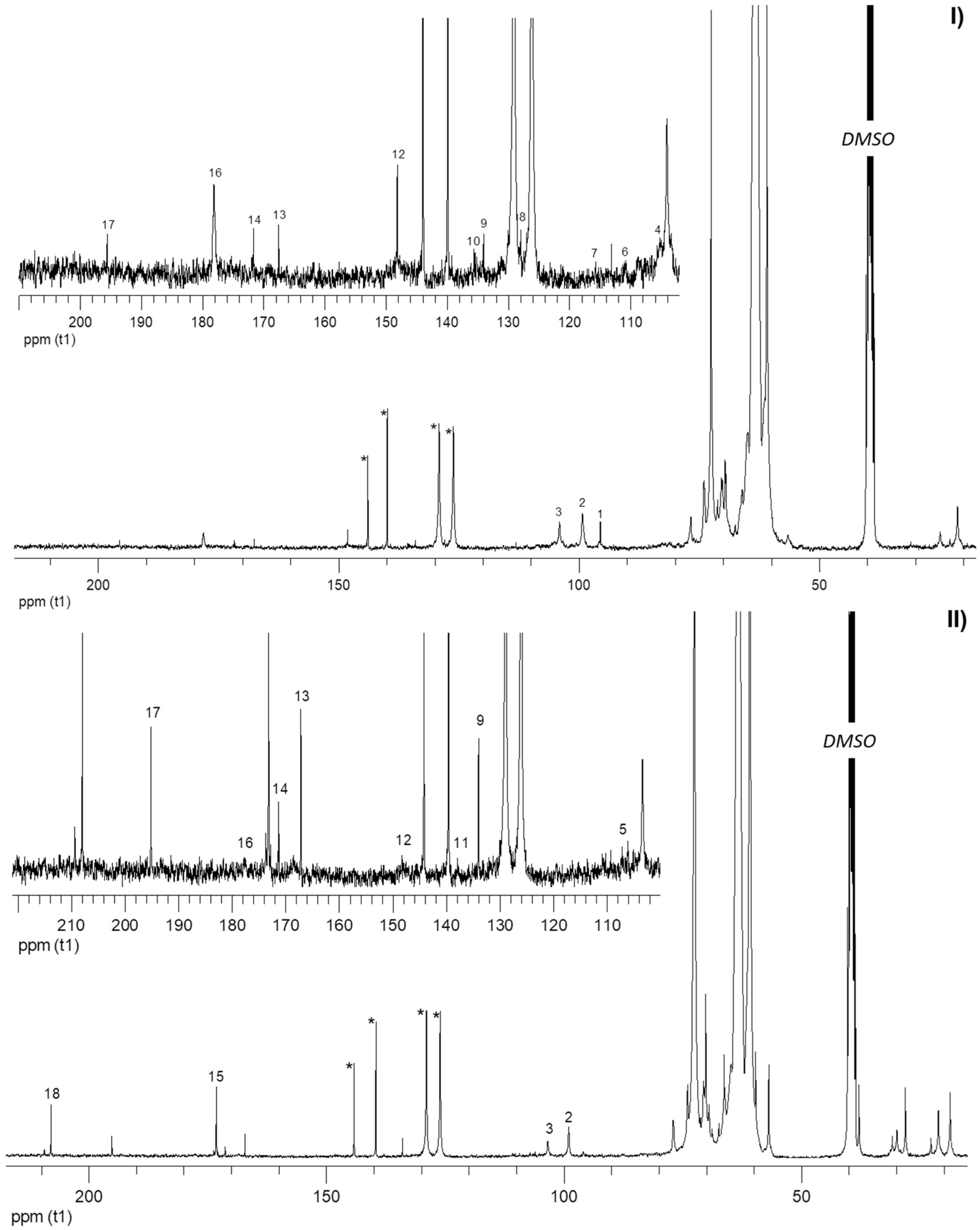

Figure S3. Qualitative ^{13}C NMR spectrum of I) W_{LGS}-15 and W_{LGS}-240; signals indicated with an asterisk (*) correspond to PTSA.
